# Ligand-Based Drug Design of Novel Antimicrobials against *Staphylococcus aureus* by Targeting Bacterial Transcription

**DOI:** 10.3390/ijms24010339

**Published:** 2022-12-25

**Authors:** Jiqing Ye, Xiao Yang, Cong Ma

**Affiliations:** 1 State Key Laboratory of Chemical Biology and Drug Discovery, Department of Applied Biology and Chemical Technology, The Hong Kong Polytechnic University, Kowloon, Hong Kong SAR, China; 2 School of Pharmacy, Inflammation and Immune Mediated Diseases Laboratory of Anhui Province, Anhui Medical University, Hefei 230032, China; 3 Department of Microbiology, Prince of Wales Hospital, The Chinese University of Hong Kong, Shatin, Hong Kong SAR, China

**Keywords:** *Staphylococcus aureus*, ligand-based drug design, pharmacophore, QSAR, virtual screening

## Abstract

*Staphylococcus aureus* is a common human commensal pathogen that causes a wide range of infectious diseases. Due to the generation of antimicrobial resistance, the pathogen becomes resistant to more and more antibiotics, resulting in methicillin-resistant *S. aureus* (MRSA) and even multidrug-resistant *S. aureus* (MDRSA), namely ‘superbugs’. This situation highlights the urgent need for novel antimicrobials. Bacterial transcription, which is responsible for bacterial RNA synthesis, is a valid but underutilized target for developing antimicrobials. Previously, we reported a novel class of antimicrobials, coined nusbiarylins, that inhibited bacterial transcription by interrupting the protein–protein interaction (PPI) between two transcription factors NusB and NusE. In this work, we developed a ligand-based workflow based on the chemical structures of nusbiarylins and their activity against *S. aureus*. The ligand-based models—including the pharmacophore model, 3D QSAR, AutoQSAR, and ADME/T calculation—were integrated and used in the following virtual screening of the ChemDiv PPI database. As a result, four compounds, including **J098-0498**, **1067-0401**, **M013-0558**, and **F186-026**, were identified as potential antimicrobials against *S. aureus*, with predicted pMIC values ranging from 3.8 to 4.2. The docking study showed that these molecules bound to NusB tightly with the binding free energy ranging from −58 to −66 kcal/mol.

## 1. Introduction

*Staphylococcus aureus* (*S. aureus*) is a common pathogen which can cause multiple infectious diseases, such as skin and soft tissue infections, pneumonia, and sepsis [1]. It is estimated that 20~40% of the general population possesses *S. aureus* in their nasal mucosa commensally [2]. Standard treatment of staphylococcal infections relays on the use of β-lactamase-stable penicillins such as flucloxacillin [3]. However, over the past years, there has been an increasing rate of methicillin-resistant *S. aureus* (MRSA) colonization and infections that have become a severe risk to global health [4]. Since its first description in 1960s, many countries experienced outbreaks of MRSA in hospitals (healthcare-associated MRSA, HA-MRSA) [5]. Subsequently, community-associated MRSA, (CA-MRSA), which was detected in individuals without previous healthcare contact, was observed in 1980s. In the mid-2000s, livestock-associated MRSA was reported [5]. With the emergence of MRSA, the treatment options become limited. In 2017, World Health Organization (WHO) published a priority list of bacteria for which novel antibiotics are urgently required [6], in which *S. aureus* was included as a pathogen with high priority to develop specific antimicrobials.

Besides antimicrobial resistance, lacking innovation in antibiotic discovery is also of concern. Since 2017, only 12 antimicrobial drugs have been approved, 10 of which belong to existing classes with known mechanisms of antimicrobial resistance [7]. Several factors are thought to be responsible for the undesirable situation for antibiotic discovery, for example, unsatisfactory revenue potential from the drug market, and challenges of structural optimization as most of current antibiotics were derived from natural products with complicated scaffolds [8]. Therefore, efficient and concerted investments in antimicrobial research are needed to accelerate and expand antimicrobial pipeline discovery.

**Figure 1:**
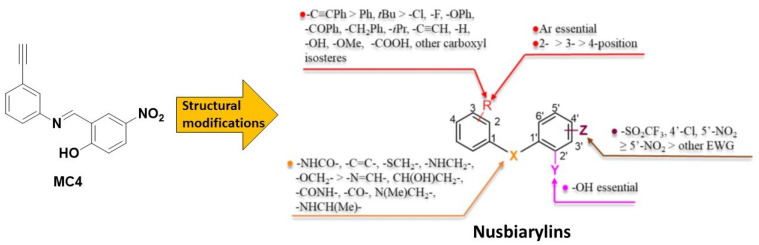
Structures of **MC4** and its derivatives (nusbiarylins), and their structure–activity relationship (SAR) against *S. aureus*.

Bacterial transcription is a crucial biological process for bacterial survival, where DNA segments are transformed into RNA molecules (mRNA, tRNA, and rRNA). This process is proceeded by RNA polymerase (RNAP) and regulated by a number of transcription factors [9]. The protein–protein interactions (PPI) between RNAP-transcription factor or factor–factor PPIs are potential targets for antimicrobial drug discovery [10]. Amongst, the PPI between two bacterial transcription factors NusB and NusE plays an important role in the formation of antitermination complex with RNAP that prevents premature transcription termination, particularly for the synthesis of rRNA [11,12,13]. Moreover, NusB and NusE are highly conserved and exclusively existing in bacteria [14,15]. A close examination of NusB-NusE PPI in the protein crystal complex revealed that the major intermolecular hydrogen bonding interactions occurred and conserved between the interacting helix of NusE and the binding groove of NusB [16,17,18]. On the structural basis of the critical contacts identified, we constructed a structure-based pharmacophore model for *in silico* screening of the mini-Maybridge compound library [19], leading to some hits, however, with no antimicrobial activity or high cytotoxicity in the preliminary study [20,21]. After refining the pharmacophore model, we discovered the first efficient NusB-NusE PPI inhibitor **MC4**, with effective inhibitory activity to NusB-NusE PPI and a minimum inhibitory concentration (MIC) against *S. aureus* at 8 µg/mL [22]. The successful development of high-throughput screening method helped us with the optimization of **MC4** [23], which resulted in the development of a series of derivatives with improved antimicrobial activity to MICs of 0.5 µg/mL [24,25,26,27], comparable to marketed antibiotic drugs. Furthermore, these compounds demonstrated excellent bioactivity to inhibit the toxin release of *S. aureus* [28]. As the target of this series of antimicrobials is NusB and these derivatives shared the common scaffold of biaryl moiety, they were named as “nusbiarylins” thereof (Figure 1).

Ligand-based drug design (LBDD) refers to a drug discovery method using a set of chemical structures of conformed ligands of a target without target structure. In the process of LBDD, molecular similarity approaches, quantitative structure–activity relationships (QSAR), and pharmacophore models are frequently used [29]. Ligand-based pharmacophore (LBP) model represents the important conformation required by identifying the largest 3D pattern of features of the inputted ligands to a target. A critical step of LBP is to identify a ‘bioactive’ conformation of an active molecule so that the remaining molecules can be aligned. Upon establishment, the model can be used to screen databases to identify possible hits. Besides LBP, QSAR is another key technique used in LBDD. Based on the hypothesis that similar structures or substituents may have comparable impacts on biological activity, correlations can be given between structures and activity. Therefore, a QSAR model can be constructed to predict the activity of new compounds from a database containing the information of structures and bioactivity. A combination of these methods was a very effective strategy in the discovery of novel drugs [30,31].

PPIs used to be considered undruggable due to large surface and flat binding site. As PPI inhibitors, the discovery and optimization of nusbiarylins has faced a series of challenges and taken a considerable time in drug design, hit identification, and lead optimization, which requested the assistance of computer-assisted calculation in each step. The similar path has been revealed in the discovery of another PPI inhibitor series, sigmacidins, designed by targeting RNAP-σ PPI [32]. On the basis of assay development [33], we have used a house-made library to identify hits [34], which were subjected to lead optimization [35,36,37,38,39]. Using a drug-like hit compound [40], we have recently improved the lead structures and antimicrobial activity [41,42,43].

With the structures of **MC4** derivatives and their activity against *S. aureus* in hand, we intended to conduct a ligand-based drug discovery workflow to discover novel antimicrobials targeting NusB-NusE PPI. Sixty-one molecules were retrieved from our previous reports [24,25,26]. Their structures were provided in the supporting information (Table S1), and their activity against *S. aureus* were displayed in Table 1. 

**Figure 2:**
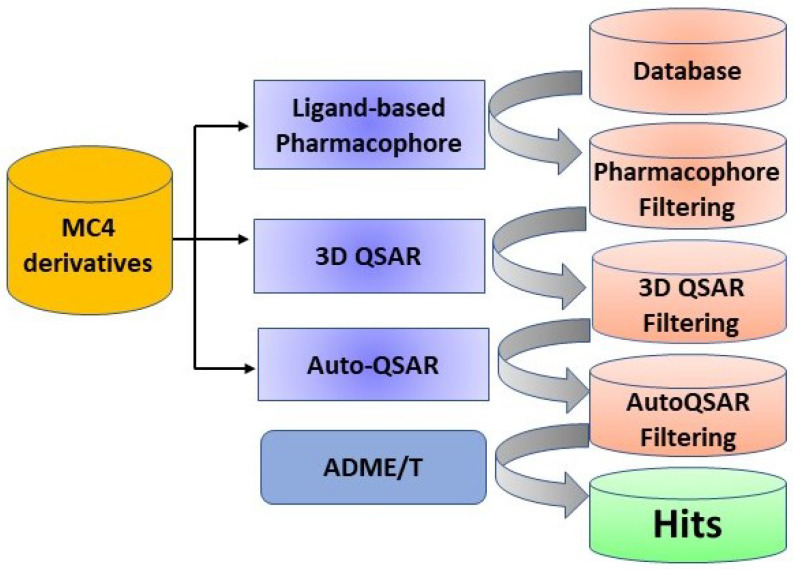
The workflow of current research.

Initially, a pharmacophore model based on **MC4** derivatives and their corresponding antimicrobial activity against *S. aureus* was constructed. Consequently, a 3D QSAR model was built to visualize how the chemical structures influence the antimicrobial activity. It was also used to predict potential antimicrobial activity of the hits identified by pharmacophore-based virtual screening. Subsequently, an AutoQSAR model was built based on machine learning methods to validate the prediction results of the 3D QSAR model. Finally, absorption, distribution, metabolism, excretion, and toxicity (ADME/T) calculations were performed to exclude the molecules with inappropriate properties. The whole protocol was summarized in Figure 2. 

## 2. Results and Discussion

### 2.1. Development of Ligand-Based Pharmacophore Hypothesis

Virtual screening, which is focused on the identification of novel hits against druggable targets, is one of the cornerstones in current drug discovery [44]. Pharmacophore-based virtual screening, including ligand-based and structure-based methods, is a highly efficient technique [45]. Nusbiarylins were validated as inhibitors of NusB-NusE PPI. In this study, LBP model was constructed in the absence of NusB or NusE. The model was generated by the PHASE module of Schrödinger Maestro 10.2. pMIC thresholds of 5.0 and 3.0 were applied to the dataset to result in eight active compounds (pMIC ≥ 5.0) and three inactive compounds (pMIC ≤ 3.0) for pharmacophore generation. As a result, 20 pharmacophore hypotheses with corresponding hypothesis scores were generated by the 11 compounds (Table 2). 

**Figure 3:**
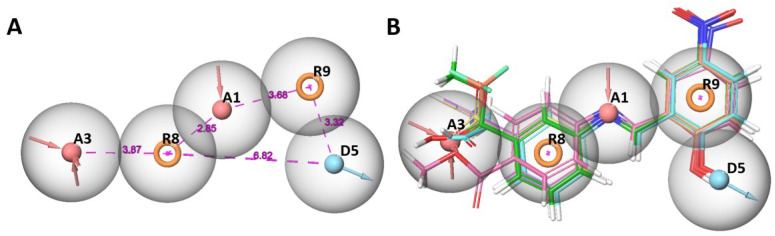
(**A**) Pharmacophore hypothesis of AADRR_1, and distances between pharmacophores; (**B**) alignment of AADRR_1 with all the active and inactive compounds.

The pharmacophore hypothesis, named AADRR_1—which comprised two acceptor (A), one donor (D) and two aromatic rings (R)—was selected for further study based on the select scores and survival scores. It has a select score of 1.608, survival score of 4.885, site score of 0.781, vector score 0.957, volume score 0.840, and BEDROC value of 0.639. The distances between the pharmacophoric features were depicted in Figure 3 showing the alignment of all the active and inactive compounds to AADRR_1.

To evaluate its ability to detect active and inactive sets of compounds, receiver operating characteristics (ROC), area under accumulation curve (AUAC), and enrichment factor (EF) were calculated. ROC reflects the sensitivity and specificity of the model and AUAC reflects the discriminated ability. When the AUAC value is 1 (100%), it indicates that active and inactive compounds can be distinguished perfectly. EF describes the number of active compounds found using a specific pharmacophore model instead of the hypothetical number of compounds found by randomly screening. In this study, AADRR_1 showed promising results with EF value of 63.00%, together with ROC value of 0.62 and AUAC value of 0.78, which confirmed that AADRR_1 represented a satisfactory model to predict active molecules.

### 2.2. Three-Dimensional QSAR 

#### 2.2.1. 3D QSAR Model

QSAR analysis is another method developed for ligand-based drug discovery for more than 50 years [46]. The first and critical step for QSAR modeling is the collection of structures and corresponding bioactivity or properties from databases or literature. The mathematical methods could then be used to describe the correlation between the structural descriptors and bioactivity (QSAR) or properties (QSPR) [47]. 

**Figure 4:**
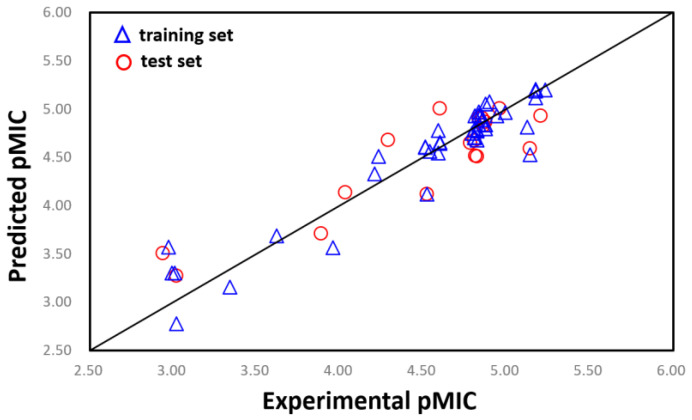
Plots of the predicted pMIC versus observed pMIC values by 3D QSAR model.

In this study, all of the 61 compounds were aligned using the Phase Hypothesis Alignment module in Maestro 10.2, then a field-based 3D QSAR model was constructed to elucidate structural features that contributed to antimicrobial activity of **MC4** derivatives. Among all the generated models, the PLS factor 3 model was selected for higher prediction accuracy compared to all other generated models with different PLS factors. Table 3 listed the statistical parameters related to the 3D QSAR model. In Maestro 10.2, r^2^ represents the non-cross-validated value for regression, r^2^cv is the LOO cross validated correlation value and Q^2^ is another non-cross-validated value which is based on the test predictions [32]. The predicted activity of the training and test sets of compounds was presented in Table 1 and the correlation with experimental activity was shown in Figure 4.

#### 2.2.2. Y-Randomization Test

Y-randomization test was used in the 3D QSAR model in order to validate and evaluate the possibility of chance correlations. Using the original independent variable matrix, several 3D QSAR were constructed using the original dependent variable matrix after dozens of random shuffles of the dependent variable (pMIC values). Results of the first 10 random shuffles for the test were shown in Table 4. The r^2^_Rand_ and r^2^cv_Rand_ values of the new models obtained after each shuffle were lower than that of original models, implying that the random correlation of the training set could be excluded.

#### 2.2.3. Contour Maps Analysis

**Figure 5:**
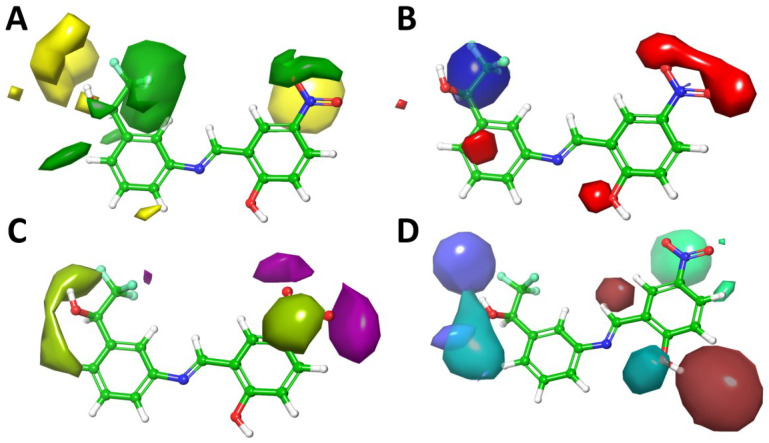
3D QSAR StDev*Coeff contour maps based on the most active compound **25**. (**A**) Steric fields: favored (green) and disfavored (yellow); (**B**) Electrostatic fields: electropositive (blue) and electronegative (red); (**C**) Hydrophobic field: favored (magenta) and disfavored (yellow green); (**D**) H-bond acceptor field: favored (cyan) and disfavored (spring green); H-bond donor field: favored (pink) and disfavored (light blue).

The colored contour maps for steric, electrostatic, hydrophobic, H-bond donor, and H-bond acceptor were generated to visualize the contribution of structural substituents to biological activity in terms of positive or negative effects (Figure 5). The most active compound **25** was used for further analysis.

In the steric contour (Figure 5A), the green regions represented the introduction of bulky substituents that might increase activity. On the contrary, the yellow regions signified the bulky groups were unfavored. It was recognized that a larger green contour was found around -CF_3_ group of compound **25**, indicating that bulky substituents might be preferred in this region, while at the opposite position around -OH, less steric hindrance would be beneficial. 

The electrostatic feature of the compounds was presented in the model as blue and red areas corresponding to the favorable and unfavorable electron-withdrawing effect in the place, respectively (Figure 5B). The contour map suggested that the -NO_2_ group could be replaced by some electron-donating groups, such as the amine group by reduction to increase the activity. 

The hydrophobic contour map was presented as magenta and yellow green (Figure 5C). The magenta-colored regions implied hydrophobic groups preferred, whereas the yellow green regions represented hydrophobic groups that were unfavorable to biological activity. The -NO_2_ group was covered by a yellow green contour with magenta cloud, indicating that it could be replaced by some hydrophilic groups with hydrophobic parts, such as alkoxyl groups and alkyl amines. In addition, the left phenyl group was roofed by another yellow green contour, indicating that some hydrophilic group could be introduced to improve the activity. 

Figure 5D depicted the effects of H-bond acceptor and donor substitutions to biological activity. The regions preferred with H-bond acceptors were represented in cyan, and the regions with H-bond acceptors disfavored were represented in spring green. Besides, H-bond donor-favorable regions were pink and unfavorable regions light blue. The cyan regions were mainly distributed near the -OH group at α position of trifluoroethyl group, indicating that oxidization of the alcohol to ketone might be beneficial to bioactivity. On the contrary, a pink contour covering the phenol group implied OH or other H-bond donor groups should be beneficial to bioactivity.

Overall, this model helped gain deep understanding of the structural information of **MC4** derivatives for further modifications. From the electrostatic and hydrophobic contour maps, we concluded that the -NO_2_ group could be replaced by other groups. Additionally, H-bond acceptor and donor contour maps demonstrated that the phenol group could be replaced by other hydrogen donors.

### 2.3. AutoQSAR

**Figure 6:**
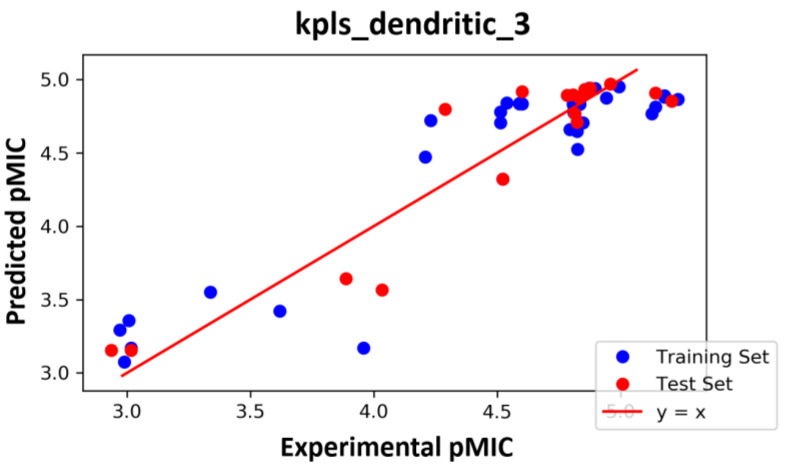
The scatter plot of observed and predicted values.

AutoQSAR uses machine learning tools along with statistical methods to generate predictive QSAR models, which were shown to be as predictive as human experts in most cases [48]. With the dataset shown in Table 1, 10 AutoQSAR models were developed (Table 5). Among these models, kpls_dendritic_3 model performed greater than the rest, with a ranking score of 0.848, R^2^ (value of R^2^ of the regression model) of 0.846, and Q^2^ value (value of Q^2^ for the predicted activities of the test set) of 0.867. We also displayed the scatter plots of observed pMIC against predicted pMIC values for model 1 in Figure 6. 

### 2.4. Pharmacophore-Based Virtual Screening

The ChemDive PPI library containing 222,447 compounds was chosen for pharmacophore-based virtual screening against the selected model AADRR_1. First of all, the PHASE database was created by applying Lipinski’s filter, which yielded a total of 355,735 conformations. Subsequently, virtual screening was conducted using the selected pharmacophore model, which identified 22,481 molecules as matches. In this study, only the compounds with the phase screen score above 1.5 and five out of five matches were chosen. This filtration led to the selection of 2,245 compounds for further screening.

### 2.5. 3D QSAR, Auto QSAR, and ADME/T Prediction

The molecules identified by pharmacophore based virtual screening were subjected to both 3D QSAR and AutoQSAR prediction. Only the compounds with pMIC values above 3.8 that predicted by both models were retained. As a result, 20 compound hits were obtained. 

**Figure 7:**
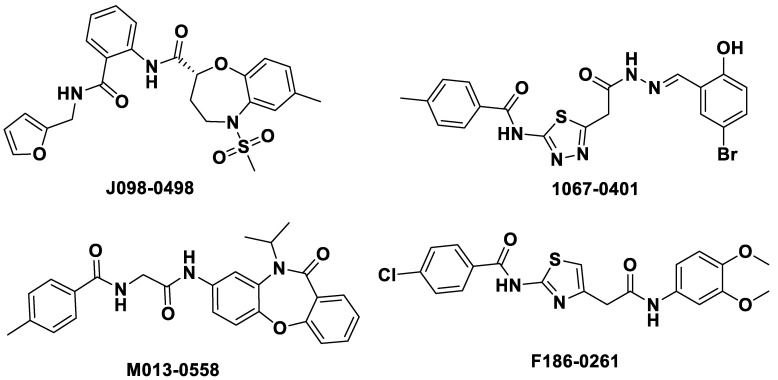
Chemical structures of the potential hit compounds.

In order to discover hits with proper drug-like properties to reduce the risk of subsequent optimization, *in silico* prediction of ADME/T properties of the hits was performed using Qikprop of Maestro 10.2 and Discovery Studio 2016 (DS 2016). Finally, four compounds, **J098-0498**, **1067-0401**, **M013-0558**, and **F186-0261** displayed acceptable parameters in the ADME/T calculation (Figure 7). 

The values of the Qikprop descriptors for the four hit compounds were depicted in Table 6 and all of them fell in the standard range of parameters [49,50]. The aqueous solubility (QPlogS) of these compounds was predicted satisfactory and the percentages of oral absorption very high (>80%). Moreover, they were expected to have great Caco-2 and MDCK cell permeability. Furthermore, their binding ability to human serum albumin (QPlogKhsa) was calculated to be moderate, indicating that more free drugs are available in circulation. In addition, CNS, #metab, and QPloghERG calculation were predicted within the standard ranges, indicating that these compounds should possess low central nervous effect, and low heart toxicity with good metabolic stability. Further pharmacokinetic and toxicology properties were calculated using DS 2016. Results revealed that all the molecules were not inhibitors or substrates of CYP2D6, indicating these molecules were less prone to induce drug–drug interactions and could be well eliminated through metabolic biotransformation. Although the four compounds were predicted to be hepatotoxic, this issue was innocuous at this stage of drug discovery, as the hit molecules would be subjected to several rounds of structural modifications for lead optimization. For the rat oral median lethal dose (LD_50_) model, the selected hits presented oral LD_50_ values ranging from 4.74 to 67.95 g/kg body weight/day, indicating that these molecules were very safe for animal experiments. 

In addition to ADME/T properties, the druglikeness parameters of the molecules were calculated using SwissADME (http://www.swissadme.ch/index.php, accessed on 13 November 2022). Results revealed that all the molecules were druggable without any violation of Lipinski’s rule of five (Table S2). 

### 2.6. Docking Studies and Binding Free Energy Calculation

#### 2.6.1. Docking Sites Prediction

**Figure 8:**
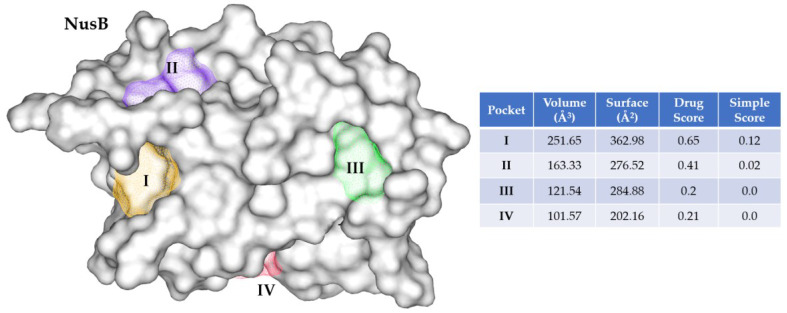
Binding pockets of NusB (PDB: 3D3B, [17]) calculated by Protein Plus.

To reveal the binding modes of the four hits with the target protein NusB, molecular docking study was performed. As there was no crystal structure of small molecule in complex with NusB, binding pockets at the surface of NusB (protein extracted from NusB:NusE complex, PDB: 3D3B, [17]) was calculated using Protein Plus (https://proteins.plus/, accessed on 13 November 2022) [51]. Results showed four pockets (I–IV) at NusB surface, while two of them (pocket I and III) located at the interface in contact with NusE (Figure 8). Moreover, compared with pocket III, pocket I possesses a relatively larger cavity with a volume of 251.65 Å^3^ and a drug score of 0.65, indicating this pocket might be more druggable. Moreover, Pocket I was composed of residues including E81, Y18, and E75 (*E. coli* amino acid residue numbering), which were exactly the designed binding site of nusbiarylins and conserved across prokaryotes [20]. Therefore, pocket I was chosen for docking study. 

#### 2.6.2. Binding Modes Analysis and Free Energy Calculation 

**Figure 9:**
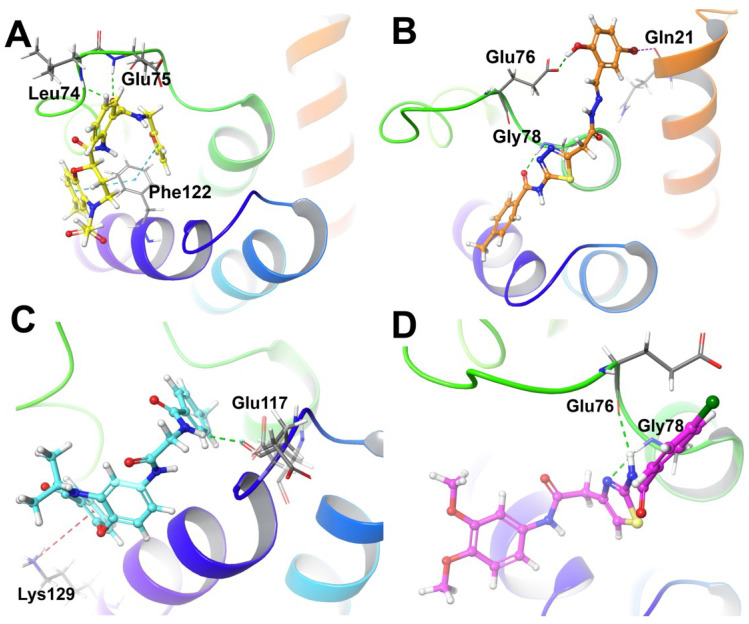
Interactions between the ligands and NusB: (**A**) **J098-0498**; (**B**) **1067-0401**; (**C**) **M013-0558**; (**D**) **F186-0261**.

The four hit compounds were docked to pocket I by Glide XP docking of Maestro 10.2. The results were shown in Table 7 and Figure 9. XP docking scores for the four compounds were ranging from −2~−3 kcal/mol, indicating that the complexes might not be stable enough. Furthermore, we calculated the binding free energy of the four hits in complex with NusB using Prime MM-GBSA module in Maestro 10.2 ($\Delta G_{MM-GBSA}^{Prime}$). Residues within 5 Å away from the ligand were set as flexible. Surprisingly, the $\Delta G_{MM-GBSA}^{Prime}$ of the four hits were around −60 kcal/mol, especially for compound **F186-0261**, with the highest $\Delta G_{MM-GBSA}^{Prime}$ value of −65.08 kcal/mol. The binding free energy implied that the hit compounds might bind to NusB tightly through a dynamic process. 

Figure 9A displayed the key interactions between **J098-0498** and NusB. The amide group of J098-0498 formed one hydrogen bond with Glu75 (O…H 2.11 Å) of NusB, and another hydrogen bond with Leu74 (O…H 2.41 Å). Additionally, both tetrahydrobenzo[*b*][1,4]oxazepane moiety and furan ring of **J098-0498** interacted with Phe122 through π–π stacking. The interactions between **1067-0401** and NusB were depicted in Figure 9B. A hydrogen bond was formed between the hydroxyl group of **1067-0401** and Glu76 (O…H 1.74 Å). Meanwhile, the hydrazide group formed another H-bond with Gly78 (O…H 1.84 Å). Indeed, these two hydrogen bonds were more stable and led to a lower binding free energy of the **1067-0401**/NusB complex when compared with that of **J098-0498**/NusB complex. In Figure 9C, **M013-0558** interacted with NusB through one hydrogen bond between the amide group of **M013-0558** (O…H 2.19 Å) and a π–cation interaction between benzene group from dibenzo[*b,f*][1,4]oxazepin-11(10*H*)-one moiety with Lys129. As shown in Figure 9D, **F186-0261** bound with NusB through two hydrogen bonds, one was formed by the thiazole moiety with Gly78 (O…H 2.41 Å) and another one by the amide moiety with Glu76 (O…H 2.11 Å). Overall, all of these compounds showed strong affinity with NusB residues including Glu75, Glu76, and Glu78 via hydrogen bonding interactions.

## 3. Materials and Methods

### 3.1. Data Preparation

All the small molecules used for model construction were collected from previous studies [24,25,26]. The unit of antimicrobial activities (MIC) in µg/mL was transformed into molarity (M). Subsequently, the values were converted to pMIC using the following equation pMIC = -lgMIC. The data set was randomly divided into training set (43 compounds) and test set (18 compounds) by Maestro 10.2. 

### 3.2. Ligand Preparation

All the molecules used in this study were minimized using Macromodel (Maestro 10.2) module with OPLS_2005 force field, and the parameters were set to default, unless mentioned otherwise. 

### 3.3. Creation of Ligand-Based Pharmacophore Model

Pharmacophore mapping was carried out using the Phase module of Maestro 10.2. The ligands were assigned as active with a threshold of pMIC ≥5.0, and inactive ≤3.0. Ligands with pMIC values between 3.0 and 5.0 were considered as moderately active. The hypothesis requirement was set to match 50% of the active ligands, number of features in the model was set to five. Six in-built pharmacophore features in the PHASE module—including the hydrogen bond donor (D), hydrogen bond acceptor (A), hydrophobic group (H), aromatic ring (R), negative ionizable group (N), and positive ionizable group (P)—were used to generate the pharmacophore model. The resulting models were scored and ranked in accordance with their survival scores, site scores, vector, and volume scores.

### 3.4. Three-Dimensional QSAR Model Construction

3D QSAR model was generated by 3D Field-based QSAR module in Maestro 10.2. The alignment was conducted by Ligand Alignment Panel. Field-based QSAR method was used to construct the 3D QSAR model. pMIC of the compounds were chosen for Activity Property [29]. The recommend field style Gaussian, including steric, electrostatic, hydrophobic, H-bond acceptor, and donor field, was selected. Maximum PLS value were set to 6 and number of ligands to leave out for cross-validation was set to 1. The aligned molecules were encompassed by a 3D cubic lattice with grid spacing of 1 Å and extending 3 Å to all sides. Finally, default energy cutoff of 30 kcal/mol for steric and electrostatic fields were used. 

### 3.5. Y-Randomization Test

An estimation of the robustness of a model is usually conducted using the Y-randomization test [52]. A new QSAR model was constructed after each iteration in which the activities of the molecules (pMIC) were randomly shuffled. Compared to the original model, the new QSARs were expected to have lower r^2^ and r^2^cv values. On the contrary, as a result of structural redundancy and chance correlation, higher r^2^ and r^2^cv mean that no acceptable 3D-QSAR can be generated for this data set.

### 3.6. Machine Learning-Based AutoQSAR Model Generation

AutoQSAR module was used to generate predictive QSAR models. All the minimized ligands were imported to Maestro workspace. The antimicrobial activity (pMIC) was set as dependent variable, while the descriptors or fingerprints related to molecular properties were computed automatically. Training/test was set to 70/30 to split the input data. Finally, top 10 predictive models were generated. These models were assessed by ranking scores, root mean square errors (RMSE), standard deviation (SD), Q^2^ and R^2^ values. 

### 3.7. Creation of Database and Pharmacophore-Based Virtual Screening

The selected pharmacophore model was used for pharmacophore-based virtual screening of a focused compounds library from ChemDiv containing 222,447 molecules. The retrieved compounds were subjected to Create Phase Database, involving ligand preparation to ionization and energy minimization, followed by ligand filtering using Lipinski’s rule. Finally, a database containing 355,735 conformations were generated. The database was screened by the selected pharmacophore model. Ligands that fulfilled five out of five matches were retained and ranked by the phase screen scores.

### 3.8. QSAR Screening and ADME/T Calculation

Compounds that selected by the pharmacophore screening were subjected to QSAR prediction. The created 3D QSAR model was imported to Field-Based QSAR module. The ligands were selected and then proceeded via the ‘Predict’ option to obtain the predicted pMIC values. On the other hand, the AutoQSAR models were imported from the AutoQSAR panel, the best model was selected to give the predictions. Finally, compounds with predicted pMIC values above 3.8 in both models were retained. ADME/T properties of the retained molecules were calculated, only compounds with parameters within standard ranges were listed as potential hit compounds.

### 3.9. Docking and Binding Free Energy Calculation 

The four ligands were prepared with LigPre module in maestro 10.2 under OPLS_2005 force field, other parameters were set default. The structure of NusB was extracted from crystal structure of NusB/NuE complex (PDB: 3D3B), which was download from PDB Data Bank. The structure was imported to maestro and prepared with Protein Preparation Wizard module. Hydrogens were added, waters were removed and the protein was minimized under OPLS_2005 force field. Subsequently, the receptor grid box was generated with using Receptor Grid Generation Module. The coordinates of the center of the docking site were defined as 7.6483, 10.4600, −2.4856 (x, y, z); and the radius of the box was 20 Å. Finally, the prepared ligands were docked to the generated grid box using Glide with XP docking methods. 

Binding free energy of the complexes was calculated using Prime MM-GBSA method under the OPLS_2005 force field use the VSGB 2.0 solvation model and residues within 5 Å away from the ligand were set as flexible. Other parameters were set default unless otherwise mentioned. 

## 4. Conclusions

In the present study, we proposed a ligand-based virtual screening approach, which integrated ligand-based pharmacophore model, 3D QSAR, AutoQSAR, and ADME/T calculation, as well as a structure-based docking study. As the result of the workflow, four compounds, including **J098-0498**, **1067-0401**, **M013-0558**, and **F186-026**, with pMIC values of 4.01 (3.81), 3.98 (4.16), 4.15 (3.83), 3.81 (3.82) predicted by 3D QSAR (AutoQSAR) models were identified as potential inhibitors of NusB-NusE PPI. Moreover, molecular docking was performed to validate the binding mode of the hits to NusB. Results showed that these compounds can potentially bind to the designed binding site at the conserved region of NusB with the binding free energies around −60 kcal/mol.

Besides NusB-NusE PPI, numerous PPIs are present in bacterial transcription such as RNAP-σ [32], RNAP-NusG PPI, and RNAP-NusA PPI [53,54,55], we believe that the workflow presented in this study will contribute to the further optimization of nusbiarylins, as well as the discovery of other novel bacterial transcription inhibitors.

## Figures and Tables

**Table 1:** Compounds selected for modeling and their measured and predicted antimicrobial activity against *S. aureus*.

Cpd.	MIC (μg/mL)	MW	MIC (M)	pMIC	3D QSAR		AutoQSAR	
					Predicted pMIC	Δ ^d^	Predicted pMIC	Δ ^d^
**1**	8	260.22	3.07 × 10^−5^	4.512	4.609	−0.096	4.780	−0.267
**2 ^a^**	4	260.22	1.54 × 10^−5^	4.813	4.516	0.297	4.767	0.046
**3**	8	260.22	3.07 × 10^−5^	4.512	4.599	−0.086	4.705	−0.192
**4**	4	256.26	1.56 × 10^−5^	4.807	4.781	0.026	4.892	−0.086
**5**	4	256.26	1.56 × 10^−5^	4.807	4.698	0.108	4.832	−0.025
**6 ^a^**	4	256.26	1.56 × 10^−5^	4.807	4.669	0.138	4.895	−0.088
**7**	4	298.34	1.34 × 10^−5^	4.873	4.834	0.038	4.936	−0.063
**8**	4	272.26	1.47 × 10^−5^	4.833	4.934	−0.102	4.858	−0.025
**9**	16	272.26	5.88 × 10^−5^	4.231	4.507	−0.276	4.721	−0.490
**10**	4	272.26	1.47 × 10^−5^	4.833	4.971	−0.138	4.863	−0.030
**11 ^a^**	4	300.27	1.33 × 10^−5^	4.875	4.862	0.013	4.941	−0.066
**12 ^b^**	2	300.27	6.66 × 10^−6^	5.176	5.203	−0.027	4.889	0.287
**13 ^b^**	2	300.27	6.66 × 10^−6^	5.176	5.183	−0.006	4.881	0.296
**14 ^a^**	8	266.25	3.00 × 10^−5^	4.522	4.119	0.403	4.322	0.200
**15**	4	272.26	1.47 × 10^−5^	4.833	4.841	−0.008	4.830	0.003
**16 ^a^**	4	272.26	1.47 × 10^−5^	4.833	4.907	−0.074	4.874	−0.041
**17 ^a^**	4	285.26	1.40 × 10^−5^	4.853	4.903	−0.050	4.932	−0.078
**18 ^a,b^**	2	321.31	6.22 × 10^−6^	5.206	4.930	0.276	4.852	0.354
**19 ^b^**	2	267.24	7.48 × 10^−6^	5.126	4.810	0.316	4.766	0.360
**20**	4	267.24	1.50 × 10^−5^	4.825	4.674	0.151	4.525	0.300
**21**	4	267.24	1.50 × 10^−5^	4.825	4.771	0.054	4.713	0.112
**22**	4	281.27	1.42 × 10^−5^	4.847	4.936	−0.089	4.705	0.142
**23 ^b^**	2	300.27	6.66 × 10^−6^	5.176	5.113	0.063	4.884	0.293
**24**	4	349.36	1.14 × 10^−5^	4.941	4.930	0.011	4.874	0.067
**25 ^b^**	2	340.25	5.88 × 10^−6^	5.231	5.195	0.035	4.865	0.366
**26**	4	315.28	1.27 × 10^−5^	4.897	5.073	−0.176	4.938	−0.042
**27 ^a^**	4	363.35	1.10 × 10^−5^	4.958	5.003	−0.045	4.969	−0.011
**28**	4	392.39	1.02 × 10^−5^	4.992	4.964	0.028	4.949	0.042
**29 ^a^**	8	318.33	2.51 × 10^−5^	4.600	5.004	−0.404	4.916	−0.316
**30**	8	318.33	2.51 × 10^−5^	4.600	4.656	−0.056	4.835	−0.235
**32**	8	318.33	2.51 × 10^−5^	4.600	4.644	−0.044	4.835	−0.235
**33**	8	266.25	3.00 × 10^−5^	4.522	4.119	0.403	4.322	0.200
**34 ^a^**	4	266.25	1.50 × 10^−5^	4.823	4.785	0.038	4.648	0.175
**35 ^a^**	4	266.25	1.50 × 10^−5^	4.823	4.506	0.318	4.708	0.115
**36**	4	242.23	1.65 × 10^−5^	4.782	4.647	0.136	4.893	−0.111
**37**	4	298.34	1.34 × 10^−5^	4.873	5.053	−0.181	4.937	−0.064
**38**	4	298.34	1.34 × 10^−5^	4.873	4.835	0.038	4.936	−0.063
**39**	4	298.34	1.34 × 10^−5^	4.873	4.794	0.079	4.940	−0.067
**40**	4	258.23	1.55 × 10^−5^	4.810	4.703	0.107	4.809	0.001
**41**	16	258.23	6.20 × 10^−5^	4.208	4.325	−0.117	4.471	−0.263
**42 ^a^**	4	258.23	1.55 × 10^−5^	4.810	4.928	−0.118	4.772	0.038
**43 ^b^**	2	276.68	7.23 × 10^−6^	5.141	4.591	0.550	4.907	0.234
**44 ^b^**	2	276.68	7.23 × 10^−6^	5.141	4.524	0.617	4.812	0.329
**45**	8	276.68	2.89 × 10^−5^	4.539	4.558	−0.019	4.842	−0.303
**46 ^a^**	8	310.23	2.58 × 10^−5^	4.589	4.774	−0.185	4.835	−0.246
**47**	16	310.23	5.16 × 10^−5^	4.288	4.679	−0.392	4.797	−0.509
**48**	8	310.23	2.58 × 10^−5^	4.589	4.545	0.044	4.838	−0.250
**49**	4	286.24	1.40 × 10^−5^	4.855	4.890	−0.035	4.910	−0.055
**50^a^**	4	248.28	1.61 × 10^−5^	4.793	4.748	0.045	4.659	0.134
**51**	4	292.29	1.37 × 10^−5^	4.864	4.826	0.037	4.923	−0.059
**52**	128	279.29	4.58 × 10^−4^	3.339	3.156	0.183	3.550	−0.212
**53 ^a,c^**	256	239.24	1.07 × 10^−3^	2.971	3.565	−0.595	3.294	−0.323
**54**	32	246.26	1.30 × 10^−4^	3.886	3.711	0.175	3.644	0.242
**55 ^a^**	256	260.29	9.84 × 10^−4^	3.007	3.299	−0.292	3.359	−0.352
**56 ^c^**	256	221.25	1.16 × 10^−3^	2.937	3.502	−0.565	3.154	−0.217
**57 ^c^**	256	250.25	1.02 × 10^−3^	2.990	3.298	−0.308	3.076	−0.086
**58 ^a^**	64	266.25	2.40 × 10^−4^	3.619	3.685	−0.066	3.423	0.196
**59**	256	266.25	9.61 × 10^−4^	3.017	3.269	−0.252	3.156	−0.139
**60 ^a^**	256	266.25	9.61 × 10^−4^	3.017	2.777	0.240	3.170	−0.153
**61**	32	345.15	9.27 × 10^−5^	4.033	4.136	−0.103	3.567	0.466

^a^ Compounds taken for the test set; compounds defined as ^b^ active (pMIC ≥ 5.0) and ^c^ inactive (pMIC ≤ 3.0) compounds selected by PHASE for pharmacophore construction; ^d^ Δ = Experimental pMIC − Predicted pMIC.

**Table 2:** Pharmacophore model generated by PHASE.

ID	HypoID	Scores					
		Select	Survival	Site	Vector	Volume	BEDROC
**1**	**AADRR_1**	1.608	4.885	0.781	0.957	0.840	0.639
**2**	**AADRR_2**	1.554	4.838	0.818	0.958	0.810	0.639
**3**	**AADRR_3**	1.527	4.821	0.841	0.950	0.805	0.629
**4**	**AAARR_1**	1.496	4.778	0.786	0.958	0.840	0.639
**5**	**AAARR_2**	1.476	4.774	0.819	0.927	0.853	0.639
**6**	**AADRR_4**	1.492	4.771	0.800	0.948	0.831	0.634
**7**	**AADRR_5**	1.584	4.769	0.674	0.985	0.827	0.615
**8**	**AADRR_6**	1.587	4.749	0.676	0.971	0.816	0.644
**9**	**AAARR_3**	1.495	4.659	0.678	0.971	0.817	0.644
**10**	**AAARR_4**	1.478	4.644	0.680	0.961	0.827	0.627
**11**	**ADRR_1**	1.208	5.004	0.998	1.000	0.895	1.000
**12**	**ADRR_2**	1.170	4.966	0.999	1.000	0.894	0.982
**13**	**AARR_1**	1.142	4.938	0.999	1.000	0.894	1.000
**14**	**ADRR_3**	1.344	4.614	0.786	0.955	0.830	0.644
**15**	**ADRR_4**	1.293	4.564	0.833	0.936	0.803	0.641
**16**	**ADRR_5**	1.330	4.547	0.731	0.955	0.832	0.614
**17**	**ADRR_6**	1.314	4.529	0.756	0.947	0.813	0.617
**18**	**ADRR_7**	1.298	4.526	0.771	0.947	0.811	0.636
**19**	**AARR_2**	1.215	4.453	0.832	0.904	0.804	0.644
**20**	**AARR_3**	1.201	4.446	0.787	0.949	0.810	0.644

**Table 3:** Statistical parameters of the selected 3D QSAR model (PLS factor 3).

SD	r^2^	r^2^ _CV_	r^2^ Scramble	Stability	F	P	RMSE	Q^2^	Pearson-r
0.212	0.895	0.756	0.502	0.943	110.6	4.05 × 10^−19^	0.29	0.792	0.895

**Table 4:** r^2^ and r^2^cv values after several Y-randomization tests.

Iteration	r^2^_Rand_	r^2^cv _Rand_	Iteration	r^2^_Rand_	r^2^cv _Rand_
**1**	0.586	0.097	**6**	0.759	0.470
**2**	0.741	0.214	**7**	0.414	−0.238
**3**	0.436	−0.202	**8**	0.727	0.323
**4**	0.701	0.121	**9**	0.728	−0.001
**5**	0.634	0.016	**10**	0.626	0.107

**Table 5:** Model report of best ten QSAR model generated by AutoQSAR.

ID	Model Code	score	S.D.	R^2^	RMSE	Q^2^	Factor
**1**	**kpls_dendritic_3**	0.848	0.251	0.846	0.232	0.867	1
**2**	**kpls_linear_3**	0.833	0.256	0.840	0.254	0.840	1
**3**	**kpls_linear_14**	0.820	0.266	0.820	0.266	0.838	1
**4**	**kpls_dendritic_14**	0.818	0.270	0.815	0.256	0.851	1
**5**	**kpls_radial_14**	0.813	0.273	0.810	0.264	0.841	1
**6**	**kpls_linear_9**	0.810	0.270	0.819	0.271	0.824	1
**7**	**kpls_radial_9**	0.804	0.282	0.803	0.277	0.816	1
**8**	**kpls_radial_44**	0.802	0.282	0.821	0.273	0.761	1
**9**	**kpls_linear_50**	0.8015	0.277	0.825	0.272	0.776	1
**10**	**kpls_molprint2D_22**	0.7957	0.279	0.823	0.275	0.764	1

**Table 6:** Results of 3D-QSAR, auto-QSAR, and ADME/T prediction.

	Parameters	J098-0498	1067-0401	M013-0558	F186-0261
**Screening**	**Phase Screen Score**	1.599	1.506	1.606	1.511
	**3D QSAR_pMIC**	4.01	3.98	4.15	3.81
	**AutoQSAR_pMIC**	3.81	4.16	3.83	3.82
**ADME/T**	**QPlogS**	−6.30	−6.22	−6.84	−6.42
	**Human Oral Absorption (%)**	96.49	81.61	100.00	100.00
	**QPPCaco**	459.59	103.39	918.22	1170.89
	**QPlogKhsa**	0.31	0.18	0.67	0.23
	**QPPMDCK**	214.947	178.206	451.12	279.964
	**#metab**	6	4	3	4
	**QPlogHERG**	−7.21	−6.75	−7.09	−6.80
	**CNS**	−2	−2	−1	−1
	**CYP2D6**	false	false	false	false
	**Hepatotoxicity**	true	true	true	true
	**Rat Oral LD_50_ (g/kg)**	67.95	4.74	9.29	5.72

**Table 7:** XP docking scores of the identified hits to NusB, and their corresponding binding free energy calculated by Prime MM-GBSA module in Maestro 10.2.

ID	XP Docking Score (kcal/mol)	$\Delta G_{MM-GBSA}^{Prime}$ (kcal/mol)
**J098-0498**	−2.63	−58.64
**1067-0401**	−2.49	−64.08
**M013-0558**	−2.98	−60.72
**F186-0261**	−2.39	−65.08

## Data Availability

Supporting information for this article is available upon request from corresponding authors.

## Supplementary Materials

The following supporting information can be downloaded at: https://www.mdpi.com/article/10.3390/ijms24010339/s1.
