# Supplementary material for: Ligand-Based Drug Design of Novel Antimicrobials against Staphylococcus aureus by Targeting Bacterial Transcription
Source: Int J Mol Sci. 2022 Dec 25;24(1):339. doi: 10.3390/ijms24010339 (PMC9820117; doi:10.3390/ijms24010339)
Supplement: Supplementary file 1 [file ijms-24-00339-s001.zip › ijms-2063138-supplementary.pdf]

Ligand-Based Drug Design of Novel Antimicrobials against *Staphylococcus aureus* by Targeting Bacterial Transcription

Jiqing Ye [1,2], Xiao Yang [3,*] and Cong Ma [1,*]

[1] State Key Laboratory of Chemical Biology and Drug Discovery, Department of Applied Biology and Chemical Technology, The Hong Kong Polytechnic University, Kowloon, Hong Kong SAR, China
[2] School of Pharmacy, Inflammation and Immune Mediated Diseases Laboratory of Anhui Province, Anhui Medical University, Hefei 230032, China
[3] Department of Microbiology, Prince of Wales Hospital, The Chinese University of Hong Kong, Shatin, Hong Kong SAR, China
* Correspondence: xiaoyang@cuhk.edu.hk (X.Y.); cong.ma@polyu.edu.hk (C.M.)

Table S1. Chemical structures for molecular modeling

Cpd.	SMILES
**1**	[O-][N+](=O)c(c1)ccc(O)c1/C=N/c2c(F)cccc2
**2** [a]	[O-][N+](=O)c(c1)ccc(O)c1/C=N/c2cc(F)ccc2
**3**	[O-][N+](=O)c(c1)ccc(O)c1/C=N/c2ccc(F)cc2
**4**	c1cccc(c1C)\N=C\c2c(O)ccc(c2)[N+]([O-])=O
**5**	Cc1cc(ccc1)\N=C\c2c(O)ccc(c2)[N+]([O-])=O
**6** [a]	Cc1ccc(cc1)\N=C\c2c(O)ccc(c2)[N+]([O-])=O
**7**	CC(C)(C)c1cc(ccc1)\N=C\c2c(O)ccc(c2)[N+]([O-])=O
**8**	[O-][N+](=O)c(c1)ccc(O)c1/C=N/c2c(OC)cccc2
**9**	[O-][N+](=O)c(c1)ccc(O)c1/C=N/c2cc(OC)ccc2
**10**	[O-][N+](=O)c(c1)ccc(O)c1/C=N/c2ccc(cc2)OC
**11** [a]	COC(=O)c1c(cccc1)\N=C\c2c(O)ccc(c2)[N+]([O-])=O
**12**	COC(=O)c1cc(ccc1)\N=C\c2c(O)ccc(c2)[N+]([O-])=O
**13**	COC(=O)c1ccc(cc1)\N=C\c2c(O)ccc(c2)[N+]([O-])=O
**14** [a]	C#Cc1cc(ccc1)\N=C\c2c(O)ccc(c2)[N+]([O-])=O
**15**	OCc1cc(ccc1)\N=C\c2c(O)ccc(c2)[N+]([O-])=O
**16** [a]	OCc1ccc(cc1)\N=C\c2c(O)ccc(c2)[N+]([O-])=O
**17** [a]	NC(=O)c1cc(ccc1)\N=C\c2c(O)ccc(c2)[N+]([O-])=O
**18** [a]	[O-][N+](=O)c(c1)ccc(O)c1/C=N/c2cc(S(=O)(=O)N)ccc2
**19**	c1cccc(c1C#N)\N=C\c2c(O)ccc(c2)[N+]([O-])=O
**20**	N#Cc1cc(ccc1)\N=C\c2c(O)ccc(c2)[N+]([O-])=O
**21**	N#Cc1ccc(cc1)\N=C\c2c(O)ccc(c2)[N+]([O-])=O
**22**	N#CCc1cc(ccc1)\N=C\c2c(O)ccc(c2)[N+]([O-])=O
**23**	O=C(O)Cc1cc(ccc1)\N=C\c2c(O)ccc(c2)[N+]([O-])=O
**24**	[O-][N+](=O)c(c1)ccc(O)c1/C=N/c(ccc2)cc2CNS(=O)(=O)C
**25**	FC(F)(F)[C@H](O)c1cc(ccc1)\N=C\c2c(O)ccc(c2)[N+]([O-])=O
**26**	CONC(=O)c1cc(ccc1)\N=C\c2c(O)ccc(c2)[N+]([O-])=O
**27** [a]	CS(=O)(=O)NC(=O)c1cc(ccc1)\N=C\c2c(O)ccc(c2)[N+]([O-])=O
**28**	CN(C)S(=O)(=O)NC(=O)c1cc(ccc1)\N=C\c2c(O)ccc(c2)[N+]([O-])=O
**29** [a]	[O-][N+](=O)c(c1)ccc(O)c1/C=N/c(cccc2)c2-c3ccccc3
**30**	[O-][N+](=O)c(c1)ccc(O)c1/C=N/c(ccc2)cc2-c3ccccc3
**32**	[O-][N+](=O)c(c1)ccc(O)c1/C=N/c(cc2)ccc2-c3ccccc3
**33**	C#Cc1cc(ccc1)\N=C\c2c(O)ccc(c2)[N+]([O-])=O
**34** [a]	c1cccc(c1C#C)\N=C\c2c(O)ccc(c2)[N+]([O-])=O
**35** [a]	C#Cc1ccc(cc1)\N=C\c2c(O)ccc(c2)[N+]([O-])=O
**36**	[O-][N+](=O)c(c1)ccc(O)c1/C=N/c2ccccc2
**37**	c1cccc(c1C(C)(C)C)\N=C\c2c(O)ccc(c2)[N+]([O-])=O
**38**	CC(C)(C)c1cc(ccc1)\N=C\c2c(O)ccc(c2)[N+]([O-])=O
**39**	CC(C)(C)c1ccc(cc1)\N=C\c2c(O)ccc(c2)[N+]([O-])=O
**40**	[O-][N+](=O)c(c1)ccc(O)c1/C=N/c2c(O)cccc2
**41**	[O-][N+](=O)c(c1)ccc(O)c1/C=N/c2cc(O)ccc2
**42** [a]	[O-][N+](=O)c(c1)ccc(O)c1/C=N/c2ccc(O)cc2
**43**	[O-][N+](=O)c(c1)ccc(O)c1/C=N/c2c(Cl)cccc2
**44**	[O-][N+](=O)c(c1)ccc(O)c1/C=N/c2cc(Cl)ccc2
**45**	[O-][N+](=O)c(c1)ccc(O)c1/C=N/c2ccc(Cl)cc2
**46** [a]	c1cccc(c1C(F)(F)F)\N=C\c2c(O)ccc(c2)[N+]([O-])=O
**47**	FC(F)(F)c1cc(ccc1)\N=C\c2c(O)ccc(c2)[N+]([O-])=O
**48**	FC(F)(F)c1ccc(cc1)\N=C\c2c(O)ccc(c2)[N+]([O-])=O
**49**	O=C(O)c1cc(ccc1)\N=C\c2c(O)ccc(c2)[N+]([O-])=O
**50** [a]	[O-][N+](=O)c(c1)ccc(O)c1/C=N/C2CCCCC2
**51**	[O-][N+](=O)c(c1)ccc(O)c1/C=N/c2cccc(c23)cccc3
**52**	C#Cc1cc(ccc1)/N=C\c(c(O)cc2)cc2C(=O)OC

**53** [a]	C#Cc1cc(ccc1)\N=C\c2c(O)ccc(F)c2
**54**	C#Cc1cc(ccc1)\N=C\c(c(O)cc2)cc2C#N
**55** [a]	C#Cc1cc(ccc1)\N=C\c2c(O)ccc(c2)CC#N
**56**	C#Cc1cc(ccc1)\N=C\c2c(O)cccc2
**57**	C#Cc1cc(ccc1)\N=C\c2cc([N+]([O-])=O)ccc2
**58** [a]	C#Cc1cc(ccc1)\N=C\c2c(O)cc([N+]([O-])=O)cc2
**59**	C#Cc1cc(ccc1)\N=C\c2c(O)c([N+]([O-])=O)ccc2
**60** [a]	C#Cc1cc(ccc1)\N=C\c2c([N+]([O-])=O)ccc(c2)O
**61**	C#Cc1cc(ccc1)\N=C\c2c(O)c(Br)cc(c2)[N+]([O-])=O

[a] Compounds taken for the test set

Table S2. Physicochemical Properties of the identified hits

ID#	J098-0498	1067-0401	M013-0558	F186-0261
**Formula**	$C_{24}H_{25}N_3O_6S$	$C_{19}H_{16}BrN_5O_3S$	$C_{26}H_{25}N_3O_4$	$C_{20}H_{18}ClN_3O_4S$
**Molecular weight**	483.54 g/mol	474.33 g/mol	443.49 g/mol	431.89 g/mol
**Num. heavy atoms**	34	29	33	29
**Num. arom. heavy atoms**	17	17	18	17
**Fraction Csp^3**	0.25	0.11	0.19	0.15
**Num. rotatable bonds**	8	8	7	9
**Num. H-bond acceptors**	6	6	4	5
**Num. H-bond donors**	2	3	2	2
**Molar Refractivity**	130.28	114.57	129.98	113.12
**TPSA**	126.33 Å^2	144.81 Å^2	87.74 Å^2	117.79 Å^2
